# Effects of neonatal hyperoxia on the critical period of postnatal development of neurochemical expressions in brain stem respiratory‐related nuclei in the rat

**DOI:** 10.14814/phy2.13627

**Published:** 2018-03-08

**Authors:** Lianwei Mu, Dong Dong Xia, Teresa Michalkiewicz, Matthew Hodges, Gary Mouradian, Girija G. Konduri, Margaret T. T. Wong‐Riley

**Affiliations:** ^1^ Departments of Cell Biology, Neurobiology and Anatomy Medical College of Wisconsin Milwaukee Wisconsin; ^2^ Department of Pediatrics Medical College of Wisconsin Milwaukee Wisconsin; ^3^ Department of Physiology Medical College of Wisconsin Milwaukee Wisconsin

**Keywords:** BDNF, brain stem, critical period, cytochrome oxidase, respiratory development, serotonin

## Abstract

We have identified a critical period of respiratory development in rats at postnatal days P12‐13, when inhibitory influence dominates and when the response to hypoxia is at its weakest. This critical period has significant implications for Sudden Infant Death Syndrome (SIDS), the cause of which remains elusive. One of the known risk factors for SIDS is prematurity. A common intervention used in premature infants is hyperoxic therapy, which, if prolonged, can alter the ventilatory response to hypoxia and induce sustained inhibition of lung alveolar growth and pulmonary remodeling. The goal of this study was to test our hypothesis that neonatal hyperoxia from postnatal day (P) 0 to P10 in rat pups perturbs the critical period by altering the normal progression of neurochemical development in brain stem respiratory‐related nuclei. An in‐depth, semiquantitative immunohistochemical study was undertaken at P10 (immediately after hyperoxia and before the critical period), P12 (during the critical period), P14 (immediately after the critical period), and P17 (a week after the cessation of hyperoxia). In agreement with our previous findings, levels of cytochrome oxidase, brain‐derived neurotrophic factor (BDNF), TrkB (BDNF receptor), and several serotonergic proteins (5‐HT_1A_ and _2A_ receptors, 5‐HT synthesizing enzyme tryptophan hydroxylase [TPH], and serotonin transporter [SERT]) all fell in several brain stem respiratory‐related nuclei during the critical period (P12) in control animals. However, in hyperoxic animals, these neurochemicals exhibited a significant fall at P14 instead. Thus, neonatal hyperoxia *delayed* but did not eliminate the critical period of postnatal development in multiple brain stem respiratory‐related nuclei, with little effect on the nonrespiratory cuneate nucleus.

## Introduction

The respiratory system in rats is not fully mature at birth. The alveoli are not fully formed in the lung (Thurlbeck 1975) and the neural control of breathing is still immature (Carroll 2003). A considerable amount of growth and maturation occurs postnatally. In the brain stem, the system passes through a brief, transient period of synaptic imbalance toward the end of the second postnatal week (P12‐13), during which excitatory synaptic activity and neurochemicals are significantly downregulated, and inhibitory neurotransmission is increased (Liu and Wong‐Riley 2002, 2005; Wong‐Riley and Liu 2005; Gao et al. 2011). At this time, the ventilatory response to acute hypoxia (10% O_2_, 5–7 min) is at its weakest (Liu et al. 2006, 2009). The expression of brain‐derived neurotrophic factor (BDNF) and its high‐affinity TrkB receptors are also transiently reduced (Liu and Wong‐Riley 2013a; Gao et al. 2014), as are the expressions of several serotonin‐ (5‐HT)‐related proteins (notably 5‐HT_1A_ and _2A_ receptors, serotonin transporter SERT, and serotonin‐synthesizing enzyme tryptophan hydroxylase or TPH) (Liu and Wong‐Riley 2008, 2010a,b). These findings have significant implications for SIDS (Sudden Infant Death Syndrome), whose cause is largely unexplained, but a brain stem 5‐HT deficiency is associated with a subset of victims (Duncan et al. 2010), and a critical period of postnatal development is one of three known risk factors (Filiano and Kinney 1994).

Prematurity remains a major risk factor for SIDS, even though the overall rate of SIDS has decreased by more than 50% since 1987 (Malloy 2013). A common therapy for premature infants is supplemental oxygen treatment to reduce apneic spells and cyanotic attacks (Bland 1980). Unfortunately, such treatment can be detrimental and often leads to retinal, pulmonary, and brain pathologies (Reich et al. 2016). In the respiratory system of rodents, neonatal hyperoxia impairs carotid body oxygen sensing function, blunts hypoxic phrenic response, reduces alveolar number and radial alveolar counts, and inhibits alveolar growth and pulmonary remodeling (Shaffer et al. 1987; Bavis et al. 2002; Kim et al. 2012; Wang et al. 2014; Teng et al. 2017).

The goal of this study was to test our hypothesis that neonatal hyperoxia perturbs the normal progression of neurochemical development in brain stem respiratory‐related nuclei and affects the critical period of brain stem development in the rat. Seven neurochemicals were assessed with immunohistochemistry followed by single neuron optical densitometry: cytochrome oxidase (a sensitive metabolic marker for neuronal activity (Wong‐Riley 1989)); BDNF, TrkB, 5‐HT_1A_ receptors, 5‐HT_2A_ receptors, SERT, and TPH. Several major respiratory‐related nuclei were chosen for assessing the effect of hyperoxia on neurochemical development: the hypoglossal nucleus (XII) is known to innervate the genioglossus muscle of the tongue and control upper airway flow by keeping the airway patent (Jordan 2001; Horner 2007); the pre‐Bötzinger complex (PBC) is one of the key centers of respiratory rhythmogenesis (Smith et al. 1991; Rekling and Feldman 1998); and the ventrolateral subnucleus of the nucleus tractus solitarius (NTS_VL_) that receives peripheral chemoafferents from the carotid body (Finley and Katz 1992) and is involved in respiratory modulation (Wasserman et al. 2000). The medullary raphé nuclei (raphé magnus [RM], raphé obscurus [ROb], and raphé pallidus [RP]) contain serotonergic neurons involved in cardiovascular regulation (Loewy and McKellar 1980) as well as respiratory regulation and presumed central chemosensitivity (Richerson 2004; Richerson et al. 2005). Likewise, serotonergic neurons in the ventrolateral medullary surface (VLMS) may contribute to central respiratory chemosensitivity (Richerson et al. 2005). The nonrespiratory cuneate nucleus (CN) is a relay in the somatosensory system with no known respiratory function and served as an internal control.

## Materials and Methods

All experiments were performed in accordance with the US National Institutes of Health Guide for the Care and Use of Laboratory Animals and approved by the Institutional Animal Care and Use Committee (IACUC) of the Medical College of Wisconsin (Milwaukee, WI).

### Animals

A total of 41 Sprague–Dawley rats of both sexes from four litters plus four dams (Harlan, Envigo, Madison, WI) were used. Two litters were exposed to 90% oxygen from the day of birth (P0) until P10, and another two litters were left in room air as controls. Both groups were then euthanized at four time points: P10 (immediately after the last day of hyperoxia at P10 and before the critical period), P12 (during the critical period), P14 (immediately after the critical period), and P17 (a week after the cessation of hyperoxia). To rule out that any neurochemical changes we might observe in the pups were due to concurrent and continuous maternal exposure to hyperoxia, we also rotated two of the dams daily to alternate nursing of the hyperoxic and normoxic groups.

The seemingly severe O_2_ level was used for the hyperoxic treatment because neonatal rats are known to be resistant to hyperoxia (Stevens and Autor 1980), unlike adult rats. Most studies use >95% O_2_ for at least the first 10 days (Juul et al. 1995), but we found that 90% O_2_ for the first 10 days in full‐term rat pups induces lung changes similar to bronchopulmonary dysplasia (BPD) in extremely premature infants (Teng et al. 2017).

### Tissue preparation for histochemistry and immunohistochemistry

Pups were anesthetized with sodium pentobarbital (60 mg/kg) i.p. and perfused intracardially with an initial warm saline followed by cold (4°C) 4% paraformaldehyde‐4% sucrose in 0.1 mol/L sodium phosphate‐buffered saline (PBS), pH 7.4. Brain stems were promptly removed, postfixed in the same fixative for 1 h at 4°C, cryoprotected with 30% sucrose in 0.1 mol/L PBS at 4°C, then frozen on dry ice and stored at −80°C until use.

Serial coronal sections (16 *μ*m thickness) of brain stems were cut with a Leica CM1850 cryostat (Leica Microsystems, Heidelberger, Nussloch, Germany). They were each processed for (1) histochemical reaction for cytochrome oxidase according to our published protocol (Wong‐Riley 1979); or (2) immunohistochemical reaction according to our published protocol (Liu and Wong‐Riley 2002) for BDNF, TrkB, 5‐HT_1A_R, 5‐HT_2A_R, SERT, and TPH. The primary antibodies used are listed in Table**** 1. Reaction products within individual neurons or the neuropil of specific brain stem nuclei were measured by semiquantitative optical densitometry using a Zeiss Zonax MPM03 photometer, a 25× objective, and a 2 *μ*m diameter measuring spot as described previously (Liu and Wong‐Riley 2002). A total of 150–350 neurons for each neurochemical within each brain stem nucleus at each age from different animals of each treatment group were measured.

**Table 1:** Primary antibodies used

Antigen	Immunogen	Manufacturer, species, type Catalog number	Dilution used
Brain‐derived neurotrophic factor (BDNF)	Human BDNF, within an internal region of BDNF	Santa Cruz Biotechnology, Inc. (Santa Cruz, CA), rabbit polyclonal IgG, sc‐546, N‐20	1:40
Tropomyosin‐related kinase B (TrkB) receptor	Intracellular C‐terminus of mouse TrkB (amino acids 794‐808	Santa Cruz Biotechnology, Inc. rabbit polyclonal IgG, sc‐12, 794	1:800
Antiserotonin receptor 1A	Amino acids 218‐336 of SR‐1A of human origin	Millipore (Chemicon), rabbit polyclonal IgG, sc‐10801(H‐119)	1:1000
SR‐2A Antibody	N‐terminal extracellular Domain of SR‐2A of Human origin	Santa Cruz Biotechnology, Inc. (Santa Cruz, CA), Mouse Monoclonal IgG, sc‐166775(A‐4),	1:100
Antiserotonin Transporter	N‐terminus/GST fusion protein (aa 1‐85)	Millipore (Chemicon), Mouse monoclonal IgG, MAB1564	1:100
Tryptophan Hydroxylase	Recombinant rabbit tryptophan hydroxylase	Sigma (St. Louis, MO), Mouse monoclonal IgG, T0678	1:300

### Data analysis and statistics

Data are presented as the mean ± SEM. Two‐way ANOVA was used to determine interactions between age and treatment groups. When significant differences were found, pairwise comparisons were made between age groups (e.g., P10 vs. P12, P12 vs. P14, and P14 vs. P17) and between treatment groups using Tukey's Studentized range test (a post hoc multiple comparisons, to control for the type I experimentwise error rate). Significance was set at *P *<* *0.01 for two‐way ANOVA and *P *<* *0.05 for Tukey's test. The *N* for statistical analyses was based on the number of animals at each age in each group, and not on the number of cells analyzed.

## Results

Hyperoxic animals had a body weight that was ~25% lower than that of controls raised in normoxia. The neurochemical trends for hyperoxic pups were comparable between those raised by the same dam throughout hyperoxia or by two different dams, each only exposed to hyperoxia every other day. Thus, the data for all hyperoxic pups at each age were combined.

The distribution of cytochrome oxidase, BDNF, TrkB, 5‐HT_1A_ receptors, 5‐HT_2A_ receptors, and SERT in XII, PBC, NTS_VL_, and CN, as well as TPH in RM, ROb, RP, and VLMS throughout the first 3 postnatal weeks in normal rats were extensively described and illustrated previously (Liu and Wong‐Riley 2002, 2003, 2005, 2008, 2010a,b, 2013a; Gao et al. 2014), and our present findings in normoxic animals were identical to those described. Thus, labeled neurons and neuropil will not be reillustrated here. The main focus of this study was to compare neurochemical development between normoxic and hyperoxic animals by means of semiquantitative optical densitometry of reaction product of these neurochemicals.

### Effects of neonatal hyperoxia on cytochrome oxidase in brain stem neurons

The expression of cytochrome oxidase (CO) was variable among neurons within each of the respiratory‐related nuclei in control, normoxic rats as described previously (Liu and Wong‐Riley 2002, 2003). Two‐way ANOVA indicated a significant difference in CO reactivity among the four time points (P10, P12, P14, and P17) in both normoxic and hyperoxic groups (*P *<* *0.01) and significant interactions between age and treatment groups (*P *<* *0.01) in each of the three respiratory‐related nuclei (XII, PBC, and NTS_VL_), but no significance was found in the nonrespiratory CN. When values from normoxic animals were compared with those of hyperoxic ones at each time point examined, the hyperoxic pups showed a significant decrease at P10 (*P *<* *0.01; Tukey's test) and a significant increase at P12 (*P <* *0.05) in XII, PBC, and NTS_VL_.

**Figure 1:**
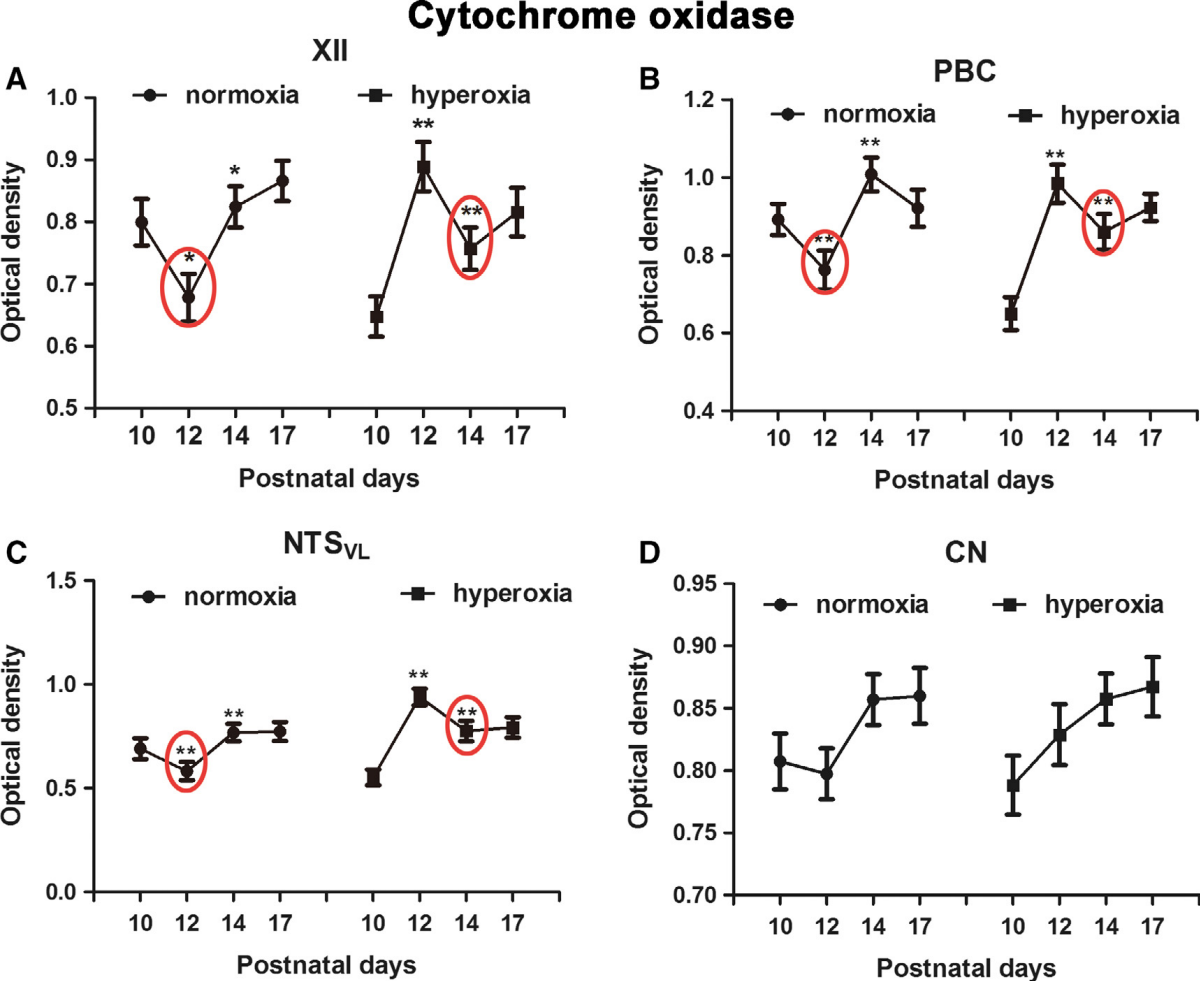
Optical densitometric values of cytochrome oxidase at the 4 developmental time points were plotted separately for the normoxic and the hyperoxic groups. A significant fall in CO reactivity at P12 and a significant rise at P14 were found in XII (A), PBC (B), and NTS_VL_ (C), but not in CN (D) of normoxic animals (left graph of each panel). In hyperoxic animals, however, the pattern was shifted to the right, such that the P10‐12‐14 pattern of normoxia became that of P12‐14‐17 of hyperoxia with a distinct fall at P14 in the latter group (right graph of each panel). *N *=* *5 for each group for this and all subsequent figures. *P <* *0.05; **P <* *0.01 (Tukey's test comparing one age group with its immediately younger age group).

However, when CO reactivity values were plotted separately for the normoxic and the hyperoxic groups, a striking pattern emerged. As shown in Figure**** 1A–**C**, the normoxic pattern for XII, PBC, and NTS_VL_ revealed a distinct decrease at P12 and a significant increase at P14 (*P <* *0.05; **P <* *0.01; Tukey's test). This is consistent with our previous report of a decrease in CO activity in multiple brain stem respiratory‐related nuclei during the critical period of respiratory development occurring at P12 in the rat (Liu and Wong‐Riley 2002, 2003). Such changes were not observed in the nonrespiratory CN (Fig. 1D). In contrast, the pattern in hyperoxic rats showed a significant decrease at P14 instead of at P12 in XII, PBC, and NTS_VL_ (*P <* *0.05; **P <* *0.01; Tukey's test). A fall was not obvious in the CN (Fig. 1D).

Thus, simply comparing normoxic and hyperoxic groups at each time point was not appropriate and could lead to erroneous conclusions, as the latter group was lagging behind in their development. The pattern of P10‐P12‐P14 in normoxic animals (high‐low‐high) is mimicked by the pattern of P12‐P14‐P17 (high‐low‐high) in hyperoxic animals. The low level at P10 in the hyperoxic group most likely reflects a low value at P8 described previously in normoxic animals, where the CO level increases from birth to P11 before falling off precipitously at P12 (Liu and Wong‐Riley 2002, 2003).

For this reason, the progression of development of all of the other neurochemicals will be analyzed separately for the normoxic group versus that of the hyperoxic group.

### Effects of neonatal hyperoxia on BDNF in brain stem neurons

Two‐way ANOVA indicated a significant difference in BDNF immunoreactivity among the four time points (P10, P12, P14, and P17) in both normoxic and hyperoxic groups (*P *<* *0.01) and significant interactions between age and treatment groups (*P *<* *0.01) in each of the three respiratory‐related nuclei (XII, PBC, and NTS_VL_), but not in the nonrespiratory CN.

**Figure 2:**
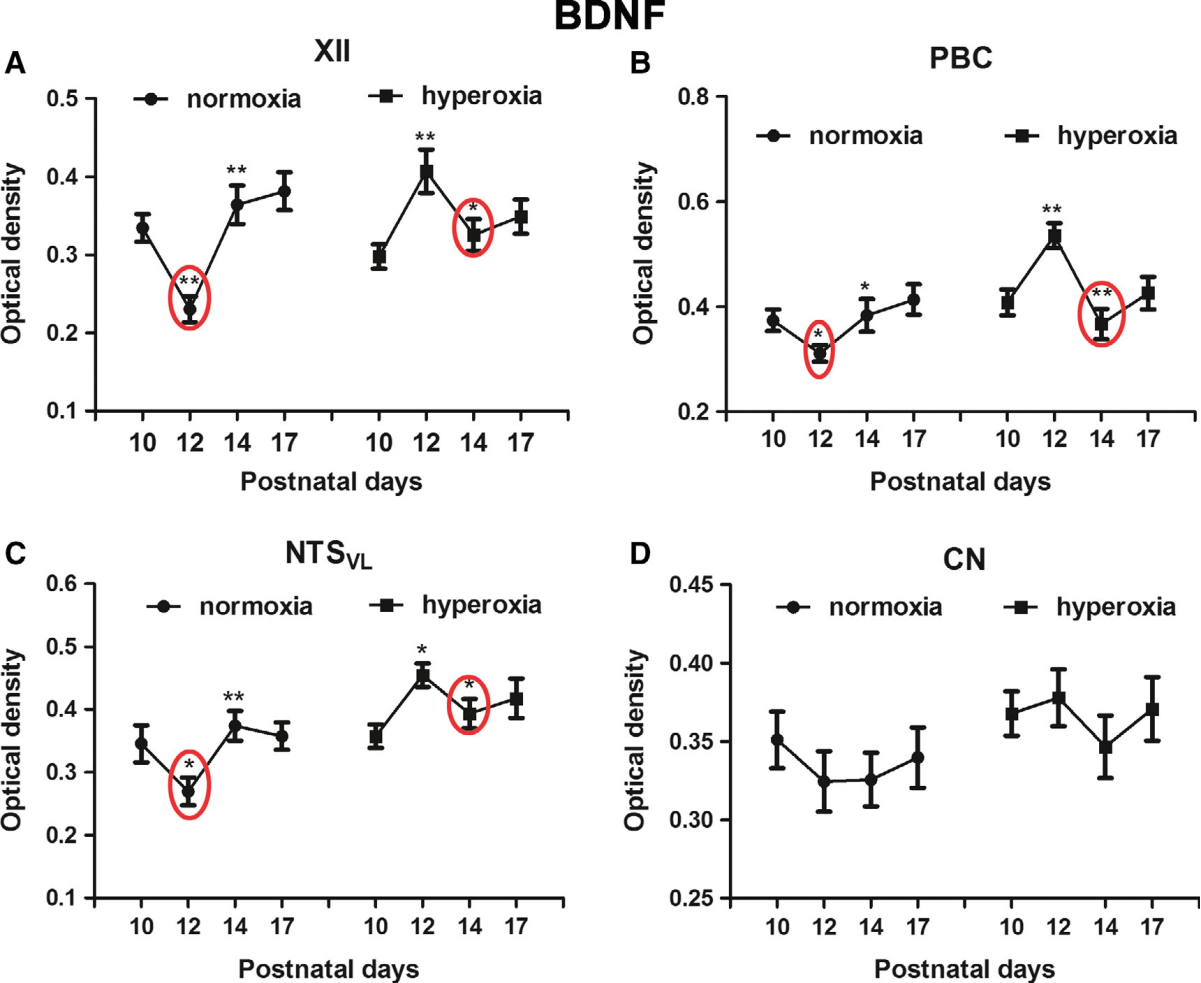
Optical densitometric measurements of immunoreaction product of BDNF in neurons of XII (A), PBC (B), NTS_VL_ (C), and CN (D) of normoxic and hyperoxic animals at P10, P12, P14, and P17. In the first three nuclei, the P10‐12‐14 pattern of normoxic animals with a distinct fall at P12 is similar to that of P12‐14‐17 in hyperoxic animals, in which the significant fall is at P14. No significant differences were found between the age groups in either normoxic or hyperoxic animals in the CN. *N *=* *5 for each group. *P <* *0.05; **P <* *0.01 (Tukey's test).

We previously described BDNF immunoreactivity in multiple brain stem respiratory‐related nuclei, including XII, PBC, and NTS_VL_ (Liu and Wong‐Riley 2013a; Gao et al. 2014). In agreement with those studies, the expression of BDNF was significantly reduced at P12 in the three respiratory‐related nuclei in normoxic animals (Fig.**** 2A–**C**; *P <* *0.05; **P <* *0.01; Tukey's test). However, in hyperoxia‐exposed animals, the decrease occurred at P14 instead of at P12 (Fig.**** 2A–**C**; *P <* *0.05; **P <* *0.01; Tukey's test).

Age‐related changes in BDNF expression was not observed in the nonrespiratory CN (Fig. 2D), which also agreed with our previous finding (Liu and Wong‐Riley 2013a). Neonatal hyperoxia also did not induce any significant changes in the CN (Fig. 2D).

### Effects of neonatal hyperoxia on TrkB in brain stem neurons

**Figure 3:**
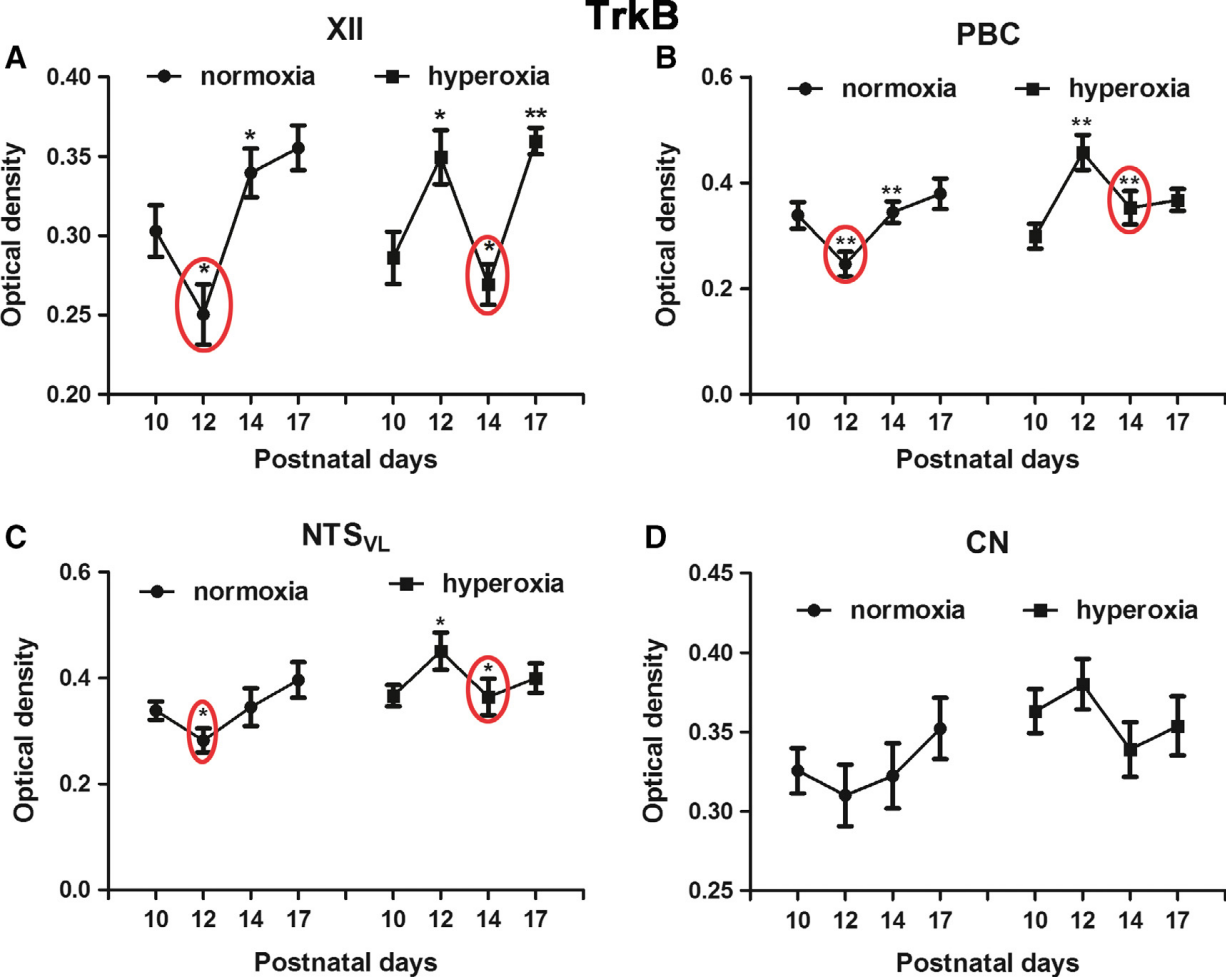
Optical densitometric measurements of immunoreaction product of TrkB in neurons of XII (A), PBC (B), NTS_VL_ (C), and CN (D) of normoxic and hyperoxic animals at P10, P12, P14, and P17. In the first three nuclei, the P10‐12‐14 pattern of normoxic animals with a distinct fall at P12 is comparable to that of P12‐14‐17 in hyperoxic animals, in which the significant fall is at P14. No significant differences were found between the age groups in either normoxic or hyperoxic animals in the CN. *N *=* *5 for each group. *P <* *0.05; **P <* *0.01 (Tukey's test).

TrkB immunoreactivity was also described in detail previously for XII, PBC, NTS_VL_, and CN (Liu and Wong‐Riley 2013a; Gao et al. 2014). Two‐way ANOVA indicated significant interactions between age and treatment groups (*P *<* *0.01) in each of the three respiratory‐related nuclei (XII, PBC, and NTS_VL_), but not in the nonrespiratory CN. Tukey's test indicated a significant reduction in TrkB expression at P12 in the three respiratory‐related nuclei (Fig.**** 3A–**C**; *P <* *0.05; **P <* *0.01), but not in CN, in normoxic animals (Fig. 3D). This is in agreement with our previous findings (Liu and Wong‐Riley 2013a; Gao et al. 2014).

With neonatal hyperoxic exposure, however, the pattern was again shifted to the right, so that the decrease occurred at P14 instead of at P12 (Fig. 3A–C; *P <* *0.05; **P <* *0.01; Tukey's test).

### Effects of neonatal hyperoxia on 5‐HT_1A_ receptors in brain stem neurons

**Figure 4:**
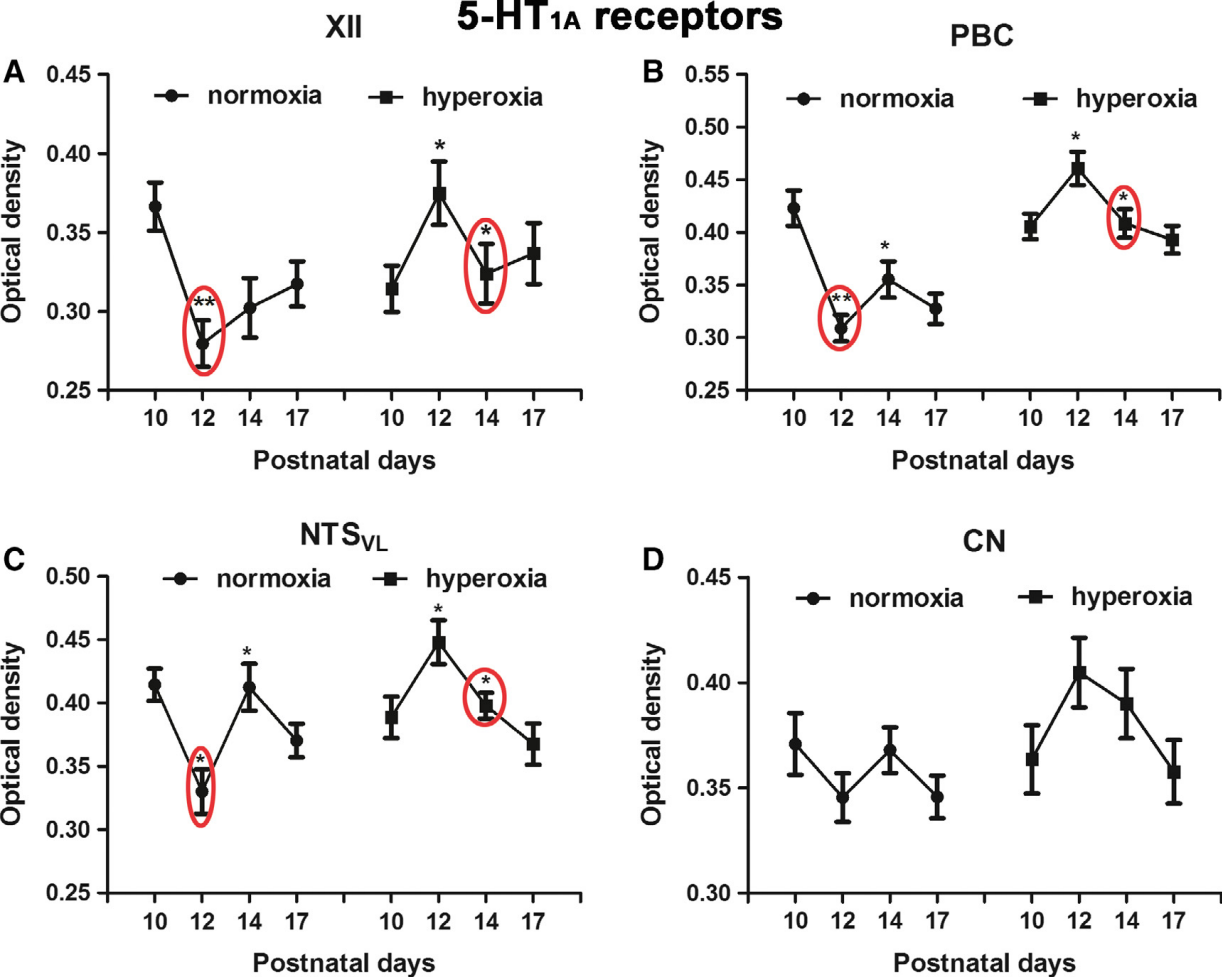
Optical densitometric measurements of immunoreaction product of 5‐HT_1A_ receptors in neurons of XII (A), PBC (B), NTS_VL_ (C), and CN (D) of normoxic and hyperoxic animals at P10, P12, P14, and P17. In the first three nuclei, the significant fall in immunoreactivity from P10 to P12 in normoxic animals is mimicked in hyperoxic animals but with a distinct fall from P12 to P14. No significant differences were found between the age groups in either normoxic or hyperoxic animals in the CN. *N *=* *5 for each group. *P <* *0.05; **P <* *0.01 (Tukey's test).

5‐HT_1A_ receptor immunoreactivity was previously reported in neurons of XII, PBC, and NTS_VL_, where their levels were high for the first postnatal week and half, before decreasing precipitously at P12 (Liu and Wong‐Riley 2010a). Two‐way ANOVA indicated significant interactions between age and treatment groups (*P *<* *0.01) in each of these three nuclei, but not in the nonrespiratory CN. Tukey's test showed a significant reduction at P12 from a relatively high level at P10 in the XII, PBC, and NTS_VL_ in normoxic animals (Fig. 4A–C; *P <* *0.05; **P <* *0.01). No significant age‐related changes were observed in the CN (Fig. 4D), in agreement with our previous report (Liu and Wong‐Riley 2010a).

**Figure 5:**
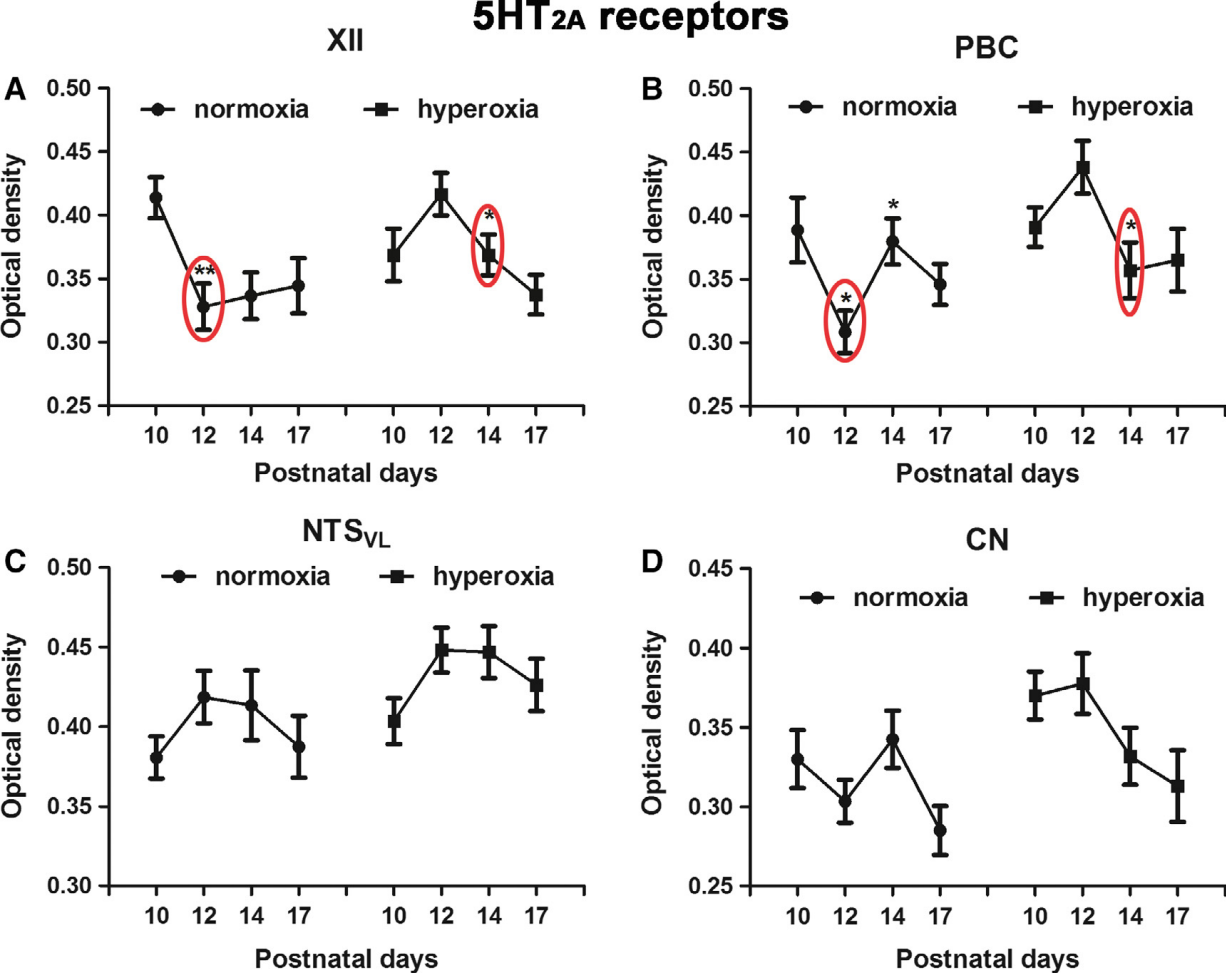
Optical densitometric measurements of immunoreaction product of 5‐HT_2A_ receptors in neurons of XII (A), PBC (B), NTS_VL_ (C), and CN (D) of normoxic and hyperoxic animals at P10, P12, P14, and P17. In the first two nuclei, a significant fall in immunoreactivity from P10 to P12 in normoxic animals is mimicked by a distinct fall from P12 to P14 in hyperoxic animals. No significant differences were found between the age groups in either normoxic or hyperoxic animals in the CN. *N *=* *5 for each group. *P <* *0.05; **P <* *0.01 (Tukey's test).

In animals exposed to neonatal hyperoxia, the decrease in 5‐HT_1A_ receptor immunoreactivity occurred at P14 instead of P12 (Fig. 5A–C; *P <* *0.05; **P <* *0.01; Tukey's test). Again, the CN did not show any significant difference among the four time points (Fig. 4D).

### Effects of neonatal hyperoxia on 5‐HT_2A_ receptors in brain stem neurons

As for 5‐HT_1A_ receptors, immunoreactivity for 5‐HT_2A_ receptors was reported previously in neurons of XII, PBC, and NTS_VL_, where their levels were high at birth and throughout the first 1.5 postnatal weeks, before falling significantly at P12 in XII and PBC, and more gradually in the NTS_VL_ (Liu and Wong‐Riley 2008, 2010a). Two‐way ANOVA indicated significant interactions between age and treatment groups (*P *<* *0.01) in XII and PBC, but not in NTS_VL_ (where changes were more gradual) (Liu and Wong‐Riley 2010a) or the nonrespiratory CN. In agreement with our previous studies, 5‐HT_2A_ receptors in normoxic animals were expressed at high levels at P10 in XII and PBC, but decreased significantly at P12 (Fig. 5A and B; *P <* *0.05; **P <* *0.01; Tukey's test).

With neonatal hyperoxic exposure, the expression of 5‐HT_2A_ receptors decreased significantly at P14 and not at P12 in XII and PBC (Fig. 5A and B; *P <* *0.05; **P <* *0.01; Tukey's test). Age‐related differences were not found for either NTS_VL_ or CN in both normoxic and hyperoxic groups (Fig. 5C and D).

### Effects of neonatal hyperoxia on SERT in brain stem neurons

**Figure 6:**
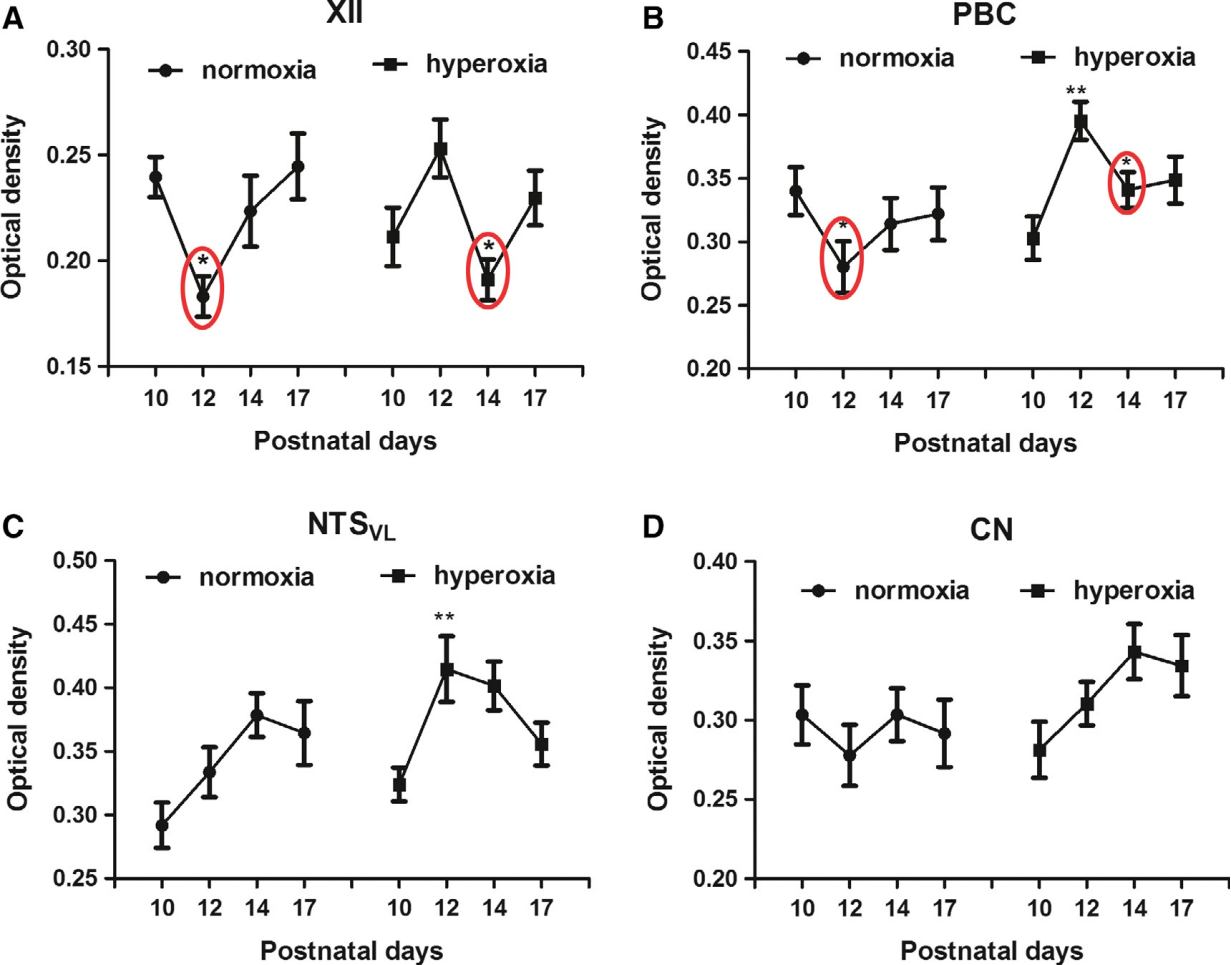
Optical densitometric measurements of immunoreaction product of SERT in the neuropil of XII (A), PBC (B), NTS_VL_ (C), and CN (D) of normoxic and hyperoxic animals at P10, P12, P14, and P17. In the first two nuclei, a significant fall in immunoreactivity from P10 to P12 in normoxic animals is paralleled by a distinct fall from P12 to P14 in hyperoxic animals. In NTS_VL_, a distinct rise in immunoreactivity was noted from P10 to P12 in hyperoxic animals. Otherwise, there were no significant differences between the age groups in NTS_VL_ of normoxic animals, nor in both groups in the CN. *N *=* *5 for each group. *P <* *0.05; **P <* *0.01 (Tukey's test).

Serotonin transporter (SERT) immunoreactivity was described previously in the neuropil of a number of brain stem respiratory‐related nuclei, including XII, PBC, and NTS_VL_, as well as in the nonrespiratory CN (Liu and Wong‐Riley 2010b). In this study, two‐way ANOVA indicated significant interactions between age and treatment groups (*P *<* *0.01) in XII and PBC, but not in NTS_VL_ (where changes were normally more gradual) (Liu and Wong‐Riley 2010b) or the nonrespiratory CN. In agreement with our previous reports, a significant reduction in the expression of SERT in the neuropil of XII and PBC was also found at P12 in normoxic animals (Fig. 6A and B; *P <* *0.05; Tukey's test). After neonatal hyperoxic exposure, the levels of SERT decreased at P14 instead of at P12 in these two nuclei (Fig. 6A and B; *P <* *0.05; Tukey's test). A significant increase at P12 was found in NTS_VL_ of hyperoxic animals but not in normoxic animals (Fig. 6C; *P <* *0.05; Tukey's test). The CN did not show any age‐related changes in either normoxic or hyperoxic animals (Fig. 6D).

### Effects of neonatal hyperoxia on tryptophan hydroxylase in brain stem neurons

**Figure 7:**
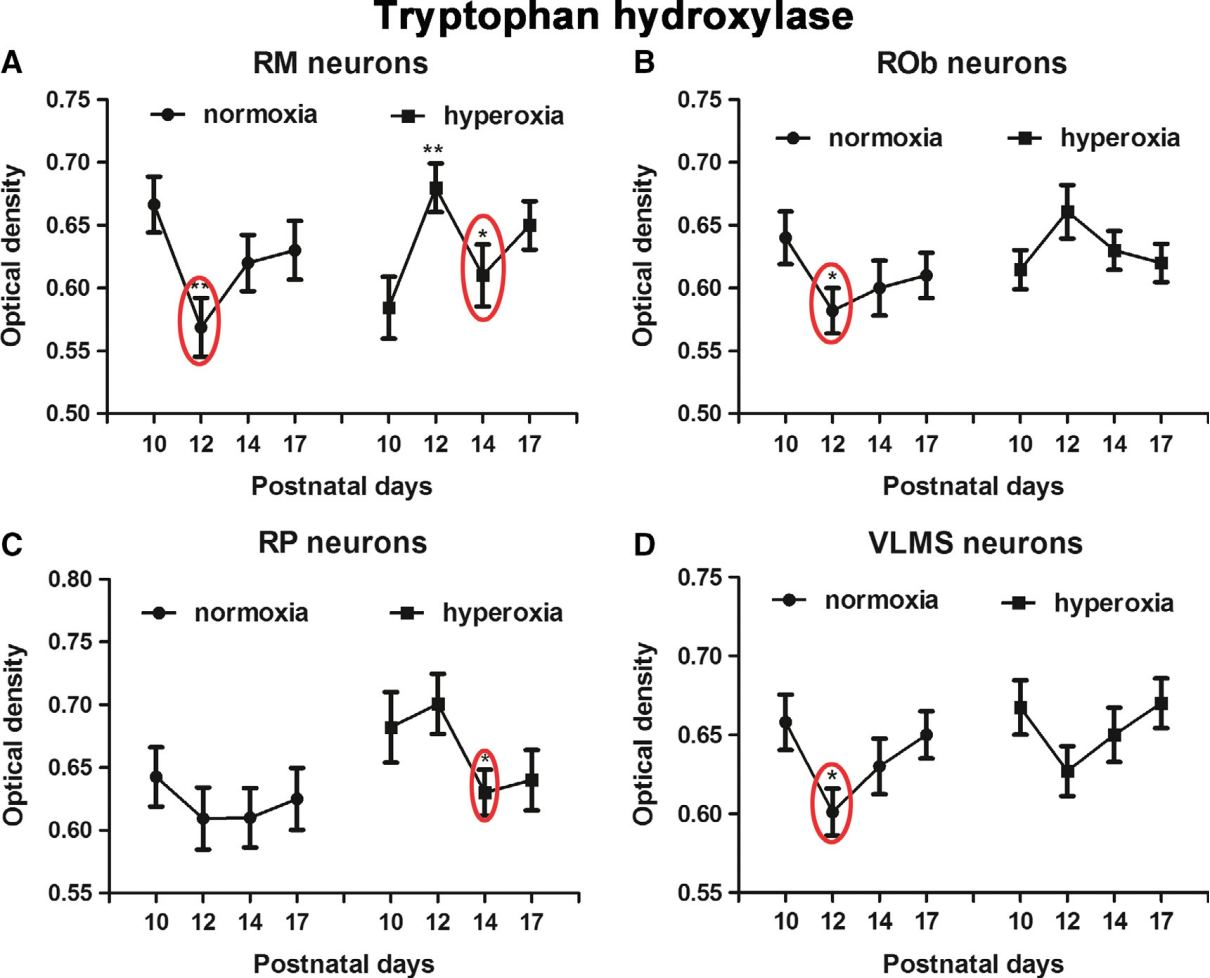
Optical densitometric measurements of immunoreaction product of tryptophan hydroxylase in neurons of RM (A), ROb (B), RP (C), and VLMS (D) of normoxic and hyperoxic animals at P10, P12, P14, and P17. In these nuclei, a distinct fall in immunoreactivity from P10 to P12 is noted in normoxic animals (though not reaching significance in the RP). This fall is shifted in hyperoxic animals to occur from P12 to P14, reaching significance in RM and RP. No significant differences were found between the age groups in ROb and VLMS neurons of hyperoxic animals. *N *=* *5 for each group. *P <* *0.05; **P <* *0.01 (Tukey's test).

Tryptophan hydroxylase is present in serotonergic neurons, and its immunoreactivity was reported in a number of brain stem nuclei, including the raphé magnus (RM), raphé obscurus (ROb), raphé pallidus (RP), and the ventrolateral medullary surface (VLMS) (Liu and Wong‐Riley 2010b). The level is relatively high for the first 1.5 postnatal weeks, before it decreased significantly at P12 (Liu and Wong‐Riley 2010b). Two‐way ANOVA indicated significant interactions between age and treatment groups for RM (*P *<* *0.01), but not for ROb (*P* value was < 0.05), RP, or VLMS. Consistent with our previous report, however, the TPH level in the current normoxic animals was high at P10, but was reduced at P12, reaching significance in RM, ROb, and VLMS, but not in RP neurons in normoxic animals (Fig. 7A–D; *P <* *0.05; **P <* *0.01; Tukey's test).

With neonatal hyperoxic exposure, the levels of TPH decreased at P14 instead of at P12 in RM, ROb and RP, reaching significance in RM and RP (Fig. 7A–C; *P <* *0.05; Tukey's test). However, the pattern was not significantly altered in the VLMS (Fig. 7D).

## Discussion

This study revealed that neonatal hyperoxia during the first 10 days of the rat's life significantly altered the development of multiple neurochemicals in a number of respiratory‐related nuclei of the brain stem, but not in the nonrespiratory somatosensory relay, the cuneate nucleus.

Hyperoxia is a common therapeutic measure for infants born prematurely in an attempt to relieve respiratory distress and to prevent the development of such ailment as the hyaline membrane disease (Cochran et al. 1965; Bland 1980). However, neonatal hyperoxia often leads to detrimental consequences, including retinal, brain, and lung pathologies demonstrable in both humans and animals (Bland 1980; Wang et al. 2014; Ramani et al. 2015; Reich et al. 2016). Alveolar growth and pulmonary remodeling are inhibited, and bronchopulmonary dysplasia is a common sequela (Shaffer et al. 1987; Warner et al. 1998; Teng et al. 2017). Neonatal hyperoxia also stunts the growth of the carotid body and induces a life‐long impairment of the hypoxic ventilatory response (Bavis et al. 1985, 2002; Donnelly et al. 2005; Wang and Bisgard 2005). As the carotid body is the key peripheral chemoreceptor that projects to the central respiratory network (Finley and Katz 1992; Gonzalez et al. 1994), the impact of neonatal hyperoxia is also intimately associated with its impact on carotid body function. Indeed, reduced body weight gain with neonatal hyperoxia (this study) is reminiscent of similar effect with carotid body denervation in the neonate (Serra et al. 2001; Liu et al. 2003). A major finding of this study, however, is that it revealed yet another form of anomaly: a disruption in the development of seven different neurochemicals important for proper functioning of neurons involved in respiratory control.

Cytochrome oxidase is the terminal enzyme of the electron transport chain responsible for the reduction of molecular oxygen to water in the process of ATP generation (Wikstrom et al. 1981). It has been proven to be a sensitive and reliable indicator of neuronal metabolic capacity and neuronal activity (Wong‐Riley 1989). In multiple brain stem respiratory‐related nuclei (including XII, PBC, and NTS_VL_), the level of CO rises with age for the first three postnatal weeks, but a distinct fall occurs at P12 in all of these nuclei, though not in the nonrespiratory CN (Liu and Wong‐Riley 2002, 2003). This indicates that the metabolic activity of the respiratory nuclei increases with the energy demand of neuronal activity during postnatal development. However, at P12‐13, there is a distinct suppression of excitatory activity and an enhancement of inhibitory activity, leading to the fall in CO activity in multiple respiratory nuclei (Liu and Wong‐Riley 2002, 2003, 2005; Gao et al. 2011). We view this narrow window as the critical period of respiratory development in the rat, as the animal's ventilatory and metabolic responses to hypoxia are the weakest at this time (Wong‐Riley and Liu 2005, 2008; Liu et al. 2006, 2009). This study found that the downregulation of CO indeed occurred at P12 in normoxic animals, but was delayed until P14 in hyperoxia‐exposed animals in the respiratory‐related nuclei XII, PBC, and NTS_VL_, but not in the CN. This indicates a developmental delay in the growth and maturation of neurons within the respiratory network.

Brain‐derived neurotrophic factor is the second neurotrophin discovered (Barde et al. 1982) and, together with its high‐affinity TrkB receptors and their signaling pathways, is involved in neuronal differentiation, migration, growth, synapse formation, and plasticity (Wardle and Poo 2003; Yoshii and Constantine‐Paton 2010). BDNF is also essential for respiratory system development (Erickson et al. 1996; Balkowiec and Katz 1998), and their disruption contribute to severe respiratory dysfunction in Rett syndrome (Ogier et al. 2007; Katz et al. 2009). Knocking out the *bdnf* gene in the mouse leads to death within the first 1‐2 postnatal weeks, most likely from respiratory complications (Erickson et al. 1996). TrkB knockout also causes early postnatal death (Klein et al. 1993). The levels of BDNF and TrkB immunoreactivity have been found to be quite high during the first 3 postnatal weeks in multiple brain stem respiratory‐related nuclei in normal rats, except for a significant reduction at P12/13 (Liu and Wong‐Riley 2013a; Gao et al. 2014). This reduction is coincidental with a fall in the expression of CO and of excitatory neurochemicals but a rise in inhibitory neurochemicals in these nuclei (Liu and Wong‐Riley 2002, 2003). It is also consistent with the fact that BDNF normally enhances excitation and suppresses inhibition (Schinder et al. 2000; Wardle and Poo 2003; Bardoni et al. 2007). Thus, a fall in BDNF/TrkB expression may contribute to the synaptic imbalance during the critical period (Liu and Wong‐Riley 2013a; Gao et al. 2014). In agreement with these findings, this study also uncovered a reduction in BDNF/TrK immunoreactivity at P12 in XII, PBC, and NTS_VL_, but not in the CN in normoxic animals. However, the reduction occurred at P14 instead in hyperoxia‐exposed animals. Thus, the downregulation of BDNF/TrkB again coincides with that of CO, both of which were delayed in hyperoxic animals.

Serotonin or 5‐hydroxytryptamine (5‐HT) is an indolamine produced mainly by raphé neurons along the midline of the brain stem (RM, ROb, and RP) and by neurons within the ventrolateral medullary surface (VLMS neurons). It exerts an early trophic effect on neurons during development and maturation, and is involved in many neural functions, among which is the modulation of cardiorespiratory function, respiratory rhythmogenesis, respiratory motoneuron excitability, upper airway reflexes, and central chemosensitivity mediated via its many receptors, chiefly the 1A and 2A receptors distributed widely throughout the brain (Haxhiu et al. 1998; Hilaire and Duron 1999; Pena and Ramirez 2002; Hodges and Richerson 2008; Cummings et al. 2009). Previously, we found that the levels of the rate‐limiting biosynthetic enzyme of serotonin (TPH), the key regulator of 5‐HT levels at the synapse (SERT), and major serotonergic receptors 1A and 2A are high during the first 1.5 postnatal weeks in many respiratory‐related nuclei of the brain stem, but a precipitous fall occurs for all of these serotonergic neurochemicals at P12, followed by either a plateau or a slight rise in these nuclei (Liu and Wong‐Riley 2008, 2010a,b). This indicates that an endogenous mechanism is in place to downregulate these neurochemicals after an initial surge in their expression. This downregulation was also found in our current normoxic animals at P12, but it was not obvious until P14 in our hyperoxia‐exposed animals.

The existence of a critical period of postnatal respiratory development in human infants has been suspected and proposed by Filiano and Kinney (1994) as one of the three major risk factors for Sudden Infant Death Syndrome. The rationale was that the peak period of SIDS was not at birth, but between the 2nd and 4th months after birth, indicating that a critical period exists postnatally. In rats, this critical period has been characterized in detail to occur toward the end of the 2nd postnatal week, specifically at P12‐P13 (reviewed in (Wong‐Riley and Liu 2008, 2005)). During this narrow window, a distinct synaptic imbalance is present, with suppressed excitation and enhanced inhibition (Liu and Wong‐Riley 2002, 2003, 2005; Gao et al. 2011) and, in addition to changes discussed above, switches in the dominance of several neurochemicals also occur at this time: from the neonatal GABA_A_ receptor *α*3 to the more mature *α*1 subunit; from the neonatal glycine receptor *α*2 and *α*3 to the more mature *α*1 subunit; from the neonatal NMDA receptor subunit 2D to the more mature 3B; and from the Cl^−^ importer NKCC1 to the Cl^−^ exporter KCC2 (Liu and Wong‐Riley 2006, 2004, 2010c, 2012, 2013b). The concurrent existence of all of these changes renders the animal more vulnerable to external stressors, and, indeed, the response to acute hypoxia is the weakest during the critical period (Liu et al. 2006, 2009). A high incidence of mortality was also noted when rat pups were exposed to sustained hypoxia from P11 to P15, and not at earlier or later times (Mayer et al. 2014).

This study showed that severe hyperoxia for the first ten postnatal days clearly caused a disruption in the development of several neurochemicals important for neuronal functioning within a number of brain stem respiratory‐related nuclei. Most notable is a significant fall in the expression of these neurochemicals at P14 instead of at P12. This strongly indicates that a) neonatal hyperoxia induces a *delay* in the neurochemical development of brain stem nuclei involved in respiration; and that b) the critical period is postponed but *not eliminated* by this treatment. The fact that the expressions of a number of these neurochemicals did not rise to a significant level from P14 to P17 in hyperoxic animals (except for TrkB in XII) as they did from P12 to P14 in normoic animals suggests that the critical period may be *prolonged* as well as delayed by hyperoxia. Significantly, carotid body denervation during postnatal development also induces a delay and a prolongation, but not the elimination, of the critical period (Liu et al. 2003). Thus, the critical period is most likely innately determined, although the timing can be modified by experimental manipulations.

Clinically, if neonatal infants are exposed to prolonged hyperoxia, it is important to bear in mind that their respiratory system may be delayed in its development, that multiple neurochemical changes may occur in their respiratory network, and that a critical period of their respiratory development needs to be anticipated at a later time point than during the normal peak period.

## Conclusions

Neonatal hyperoxia is a common therapy for prematurity, a known risk factor for SIDS, the cause of which remains elusive but its peak is during a critical period of postnatal development. This study found that severe hyperoxia for the first 10 postnatal days in the rat disrupted the development of seven neurochemicals in three brain stem respiratory‐related nuclei, but not in the nonrespiratory cuneate nucleus. Significantly, the expression of these neurochemicals fell at P14 in hyperoxic animals instead of at P12, the critical period in normoxic animals. Thus, neonatal hyperoxia appeared to *delay* and perhaps prolong but did *not eliminate* the critical period, suggesting that the critical period is genetically determined, and only the timing can be altered. Prolonged neonatal hyperoxia is detrimental to the development of the respiratory system. The effect of neonatal hyperoxia on long‐term development and maturation of neurochemicals awaits future investigation.

## Conflict of Interest

All authors have no conflict of interest to declare.
